# Association between transitional care factors and hospital readmission after transcatheter aortic valve replacement: a retrospective observational cohort study

**DOI:** 10.1186/s12872-019-1003-9

**Published:** 2019-01-18

**Authors:** Andrew Czarnecki, Peter C. Austin, Stephen E. Fremes, Jack V. Tu, Harindra C. Wijeysundera, Dennis T. Ko

**Affiliations:** 1 Schulich Heart Centre, Division of Cardiology, Sunnybrook Health Sciences Centre, 2075 Bayview Ave, Toronto, Ontario M4N 3M5 Canada; 2 Department of Medicine, University of Toronto, Suite RFE 3-805, 200 Elizabeth Street, Toronto, Ontario M5G 2C4 Canada; 3 Institute of Health Policy, Management and Evaluation, University of Toronto, 155 College Street, Suite 425, Toronto, Ontario M5T 3M6 Canada; 4 Institute for Clinical Evaluative Sciences, G-106 2075 Bayview Ave, Toronto, Ontario M4N 3M5 Canada

**Keywords:** Transcatheter aortic valve replacement, Aortic stenosis, Hospital readmission, Transition of care

## Abstract

**Background:**

Studies have shown that patients who undergo trans-catheter aortic valve replacement (TAVR) have high rates of hospital readmission. Our objectives were to identify the causes of readmission after TAVR, determine whether transitional care factors were associated with a reduction in readmission and to identify other predictors that could be used to target quality improvement efforts.

**Methods:**

We conducted a chart abstraction study that included all patients who underwent TAVR in Ontario, Canada between 2007 and 2013 and survived to hospital discharge. These data were linked to provincial administrative databases. The association between transitional care factors (home care, rehabilitation, family physician and cardiologist follow-up) and 1-year hospital readmission was examined using a time-to-event analysis. Cause-specific hazards models were used to account for the competing risk of death.

**Results:**

There were 937 patients in the cohort and the rate of readmission at 1-year was 49%. The most common causes of readmission were heart failure and bleeding. Rehabilitation (HR 1.34, 95% CI 1.11–1.62; *p* = 0.002) and cardiologist follow-up (HR 1.41, 95% CI 1.14–1.75; *p* = 0.002) were both associated with higher readmission rates. While, home care (HR 1.18, 95% CI 0.96–1.44; *p* = 0.12) and family physician follow-up (HR 1.04, 95% CI 0.85–1.28; *p* = 0.71) were not associated with readmission.

**Conclusion:**

Readmission post TAVR is common; however, we did not identify any transitional care factors associated with reductions in hospital readmission. This suggests ongoing research is required to identify targets for improvement in post-procedural care.

## Background

Transcatheter aortic valve replacement (TAVR) has revolutionized the treatment of elderly and high-risk patients with aortic stenosis (AS), as the number of patients with AS continue to rise [1]. However, recent studies have shown that hospital readmission after TAVR is very common with rates at 30-days ranging from 15 to 21% and 1-year rates are as high as 53% [2, 3]. While the goal of TAVR is to alter disease trajectory, these data suggest that despite treatment of AS these patients continue to be high utilizers of healthcare resources.

Despite expanding indications and rising volumes, the cause of hospital readmission after TAVR is still poorly understood and modifiable factors that could be used to design targeted interventions to reduce these hospitalizations have not be identified. While transitional care factors, such as physician follow-up, have been associated with lower readmission rates in other disease states, the impact of these factors in the TAVR population is unknown [4–8]. Accordingly, our objectives were to identify the causes of hospital readmission within 1 year of TAVR, to determine whether transitional care factors are associated with a reduction in readmission, and to identify any modifiable determinants that could be used as targets for improvement.

## Methods

A retrospective observational cohort study was performed using chart abstraction data linked to administrative datasets held at the Institute of Clinical Evaluative Sciences (ICES), Toronto, Ontario, Canada.

### Data sources

We conducted a chart abstraction study in Ontario to capture all TAVR procedures performed from 2007 to 2013 [9, 10]. The database included detailed demographic, clinical and procedural data. Data from the clinical database were linked using unique encoded identifiers to administrative databases and analyzed at ICES to protect patient confidentiality. This comprehensive chart abstraction was performed by six trained nurse abstractors using standardized data definitions.

The Canadian Institutes for Health Information Discharge Abstract Database was used to identify hospital readmissions and comorbidities. Validated disease state definitions were used to establish comorbidities including diabetes, hypertension, and heart failure [11–13]. Frailty was identified based on John Hopkins Ambulatory Clinical Groups software which uses a proprietary set of diagnostic codes found in administrative data [14]. The Ontario Health Insurance Plan and the ICES Physician Database were used to identify physician visits and their specialty, respectively. The Home Care Database and the National Rehabilitation Database facilitated identification of home care services and use of in-patient rehabilitation. Finally, the Registered Persons Database was used to assess vital status.

### Cohort

All patients who underwent TAVR in Ontario between April 1st, 2007 and March 31st, 2013 were included. Patients were excluded if they died prior to discharge during the index hospitalization. All patients had a minimum follow-up of 1 year.

### Outcomes

The primary outcome was all-cause readmission within 1 year of discharge from the index TAVR hospitalization.

### Transitional care factors

To evaluate the association between transitional care interventions and hospital readmission after TAVR, we assessed the following factors: follow-up with a family physician, follow-up with a cardiologist, use of home care services and in-patient rehabilitation. Home care services were considered to be present if any in-home service was delivered after discharge and includes: nurse visitation, physiotherapy, occupational therapy, personal support worker visits, as well as homemaking services. Due to the inability to accurately determine whether a physician visit occurred during hospitalization or as an out-patient, visits that occurred on the same day as hospital admission were excluded.

### Statistical analysis

Patient characteristics were compared based on the presence or absence of readmission within 1 year. Standard descriptive statistics were used. Causes of readmission were characterized based on aggregate diagnoses using groupings of ICD-10 codes [9].

Cause-specific proportional hazards models were used for modeling the hazard of readmission, treating death prior to readmission as a competing risk (known to be 20% at 1 year) [3]. This approach models the instantaneous hazard of an of event of interest in subjects who are currently event-free (i.e, who are alive and have not yet been readmitted to hospital). This strategy is appropriate when studying ‘etiologic’ associations [15]. Variables for inclusion in the regression model were chosen ‘a priori’, based on the clinical knowledge of content experts and prior literature [2, 16–18]. The following variables were included: demographics (age, sex, living status), clinical characteristics [frailty, New York Heart Association (NYHA) functional class, left ventricular function], comorbidities (diabetes, dementia, heart failure, myocardial infarction, atrial fibrillation, lung disease, cerebrovascular disease, dialysis, peripheral vascular disease, liver disease, peptic ulcer disease, bleeding), previous cardiac interventions [percutaneous coronary intervention (PCI), coronary-aortic bypass surgery], lab values [hemoglobin, estimated glomerular filtration rate (eGFR)], prior health care utilization (hospitalization 30 days prior to TAVR), procedural factors (valve-in-valve, elective vs urgent, vascular access site, self-expandable vs balloon expandable valve), post-procedural complications (permanent pacemaker, bleeding/vascular complication/transfusion, stroke, delirium), post-TAVR echocardiographic findings (aortic regurgitation, mitral regurgitation), transitional care factors and year of the TAVR procedure. Collinearity was assessed using variance inflation factors. Time to first readmission was modelled in all analyses. Patients were censored at maximum follow-up or if death occurred before readmission. Transitional care factors were included in the regression model as time-varying covariates. Robust sandwich variance estimates were used to account for clustering of patients within hospitals. The incidence of readmission and death over time were estimated using cumulative incidence function (CIF) curves.

Two sensitivity analyses were performed. First, due to the concern that factors such as physician follow-up were in the causal pathway for readmission and to better understand the influence of transitional care factors, we performed a landmark analysis assessing outcomes occurring between day 31 and day 365. In this analysis, patients who were readmitted or died within the first 30 days were excluded. Transitional care factors were operationalized in a dichotomous fashion based on their occurrence within the first 30 days. Secondly, due to concern that early TAVR experience may not be representative of current practice, we selected a cohort of patients who underwent TAVR after 2010 for separate analysis. The primary analysis was repeated in this sub-cohort to ensure overall consistency in our results.

All analyses were performed using SAS statistical software, version 9.3 (SAS Institute, Cary, NC). A 2-tailed *p* value of < 0.05 was considered significant.

## Results

### Baseline characteristics

**Fig. 1:**
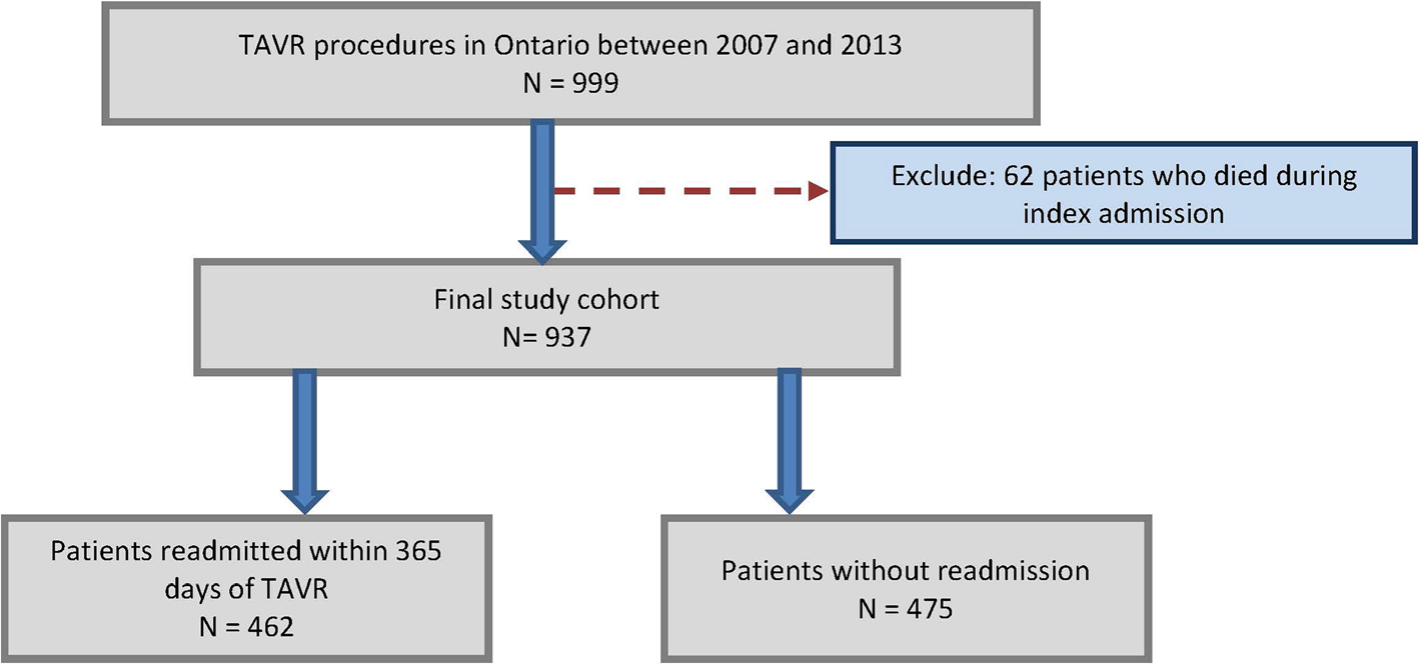
Study cohort creation

There were 999 patients who underwent TAVR between 2007 and 2013. After excluding 62 patients (6.2%) who did not survive to hospital discharge, our final study cohort included 937 patients (see Fig. 1). At least 1 year follow-up was available for all patients and mean follow-up was 2.6 +/− 1.4 years (standard deviation). There were 462 patients readmitted within 1-year of the index hospitalization, representing 49.3% of the cohort. Among those readmitted, median time to readmission was 64 days (IQR 17–157). Patients readmitted had higher baseline NYHA class and a trend towards more frailty (Table 1). There were large baseline differences in preexisting cardiac conditions, including atrial fibrillation (37.9% vs 26.1%, *p* < 0.001), heart failure (50.2% vs 37.7%, p < 0.001) and prior PCI (35.9% vs 28.4%, *p* = 0.01), all higher in readmitted patients. Patients who were readmitted had higher rates of lung disease (22.7% vs 12.6%, *p* < 0.001) and bleeding history (29.9% vs 18.9%, p < 0.001).

**Table 1:** Baseline characteristics of patients readmitted within 1 year

Characteristic	Readmitted	Not Readmitted	Overall	*p* value
	*N* = 462	*N* = 475	*N* = 937	
Demographics				
Age, median (IQR)	84 (79–87)	83 (78–87)	83 (78–87)	0.14
Female	204 (44.2%)	211 (44.4%)	415 (44.3%)	0.94
Baseline living status				
Independent	366 (79.2%)	400 (84.2%)	766 (81.8%)	0.10
Dependent	87 (18.8%)	65 (13.7%)	152 (16.2%)	
Cardiac Risk Factors				
Diabetes	230 (49.8%)	224 (47.2%)	454 (48.5%)	0.42
Dyslipidemia	330 (71.4%)	326 (68.6%)	656 (70.0%)	0.35
Hypertension	448 (97.0%)	448 (94.3%)	896 (95.6%)	0.05
Clinical Characteristics				
NYHA Class				
I or II	64 (13.9%)	82 (17.3%)	146 (15.6%)	0.01
III	290 (62.8%)	308 (64.8%)	598 (63.8%)	
IV	83 (18.0%)	52 (10.9%)	135 (14.4%)	
Frailty	70 (15.2%)	54 (11.4%)	124 (13.2%)	0.09
Weight (kg), median (IQR)	72 (62–82)	73 (62–84)	72 (62–83)	0.30
Cardiac comorbidities and interventions				
Atrial fibrillation	175 (37.9%)	124 (26.1%)	299 (31.9%)	< 0.001
Prior heart failure	232 (50.2%)	179 (37.7%)	411 (43.9%)	< 0.001
Prior myocardial infarction	115 (24.9%)	94 (19.8%)	209 (22.3%)	0.06
Prior PCI	166 (35.9%)	135 (28.4%)	301 (32.1%)	0.01
Prior CABG	133 (28.8%)	172 (36.2%)	305 (32.6%)	0.02
Prior AVR	35 (7.6%)	40 (8.4%)	75 (8.0%)	0.63
Medical comorbidities				
Cerebrovascular disease	38 (8.2%)	38 (8.0%)	76 (8.1%)	0.90
Peripheral vascular disease	92 (19.9%)	74 (15.6%)	166 (17.7%)	0.08
Dementia	12 (2.6%)	8 (1.7%)	20 (2.1%)	0.33
Dialysis	26 (5.6%)	6 (1.3%)	32 (3.4%)	< 0.001
Lung disease	105 (22.7%)	60 (12.6%)	165 (17.6%)	< 0.001
Cancer	67 (14.5%)	50 (10.5%)	117 (12.5%)	0.07
Liver disease	10 (2.2%)	6 (1.3%)	16 (1.7%)	0.29
Peptic ulcer disease	34 (7.4%)	12 (2.5%)	46 (4.9%)	< 0.001
Prior bleeding history	138 (29.9%)	90 (18.9%)	228 (24.3%)	< 0.001
Laboratory markers				
eGFR, median (IQR)	54 (39–70)	62 (46–78)	58 (42–74)	< 0.001
Hemoglobin (g/L), median (IQR)	117 (105–129)	124 (113–134)	120 (109–132)	< 0.001
Echocardiographic findings				
Aortic valve area (cm2), mean ± SD	0.68 ± 0.22	0.70 ± 0.24	0.69 ± 0.23	0.16
Mean AoV (mmHg), mean ± SD	46 ± 15	46 ± 16	46 ± 16	0.80
Left ventricular dysfunction	120 (26.0%)	135 (28.4%)	255 (27.2%)	0.42
Composite measures				
EuroScore II (%), mean ± SD	0.07 ± 0.07	0.07 ± 0.05	0.07 ± 0.06	0.06
Charlson Score, mean ± SD	2.72 ± 2.19	1.97 ± 1.91	2.34 ± 2.09	< 0.001
Hospitalization 30 days before TAVR	147 (31.8%)	116 (24.4%)	263 (28.1%)	0.01
Transitional care factors (≤30 days)				
In-patient rehabilitation	79 (17.1%)	41 (8.6%)	120 (12.8%)	< 0.001
Home care utilization	204 (44.2%)	167 (35.2%)	371 (39.6%)	0.005
Family physician follow-up	382 (82.7%)	405 (85.3%)	787 (84.0%)	0.28
Cardiologist follow-up	303 (65.6%)	258 (54.3%)	561 (59.9%)	< 0.001

Abbreviations: *IQR* interquartile range, *NYHA* New York Heart Association, *PCI* percutaneous coronary intervention, *CABG* coronary-aorto bypass grafting, *AVR* aortic valve replacement, *eGFR* estimated glomerular filtration rate, *AoV* aortic valve, *SD* standard deviation

In terms of transitional care factors, in-patient rehabilitation (17.1% vs 8.6%; p < 0.001) and home care utilization (44.2% vs 35.2%; *p* = 0.005) within the first 30 days were both more frequent in patients readmitted. There was no difference in family physician follow-up, but cardiologist follow-up was more common in patients readmitted (65.6% vs 54.3%; *p* < 0.001). Family physician follow-up occurred at a median of 7 days post discharge (IQR 3–17 days) while cardiologist follow-up occurred at median of 26 days (IQR 11–41 days).

### Procedural characteristics, complications and outcomes

There was no difference in procedural urgency between patients with and without hospital readmission (Table 2). Non-trans-femoral vascular access was more common in patients readmitted (27.9% vs 22.9%, *p* = 0.05). Procedural complications including delirium (11.7% vs 5.5%) and bleeding/transfusion/vascular complications (38.1% vs 25.7%) were significantly more prevalent in readmitted patients.

**Table 2:** Procedural characteristics, complications and echocardiographic findings of patients readmitted within 1 year

Characteristic	Readmitted	Not Readmitted	Overall	*p* value
	*N* = 462	*N* = 475	*N* = 937	
Procedural characteristics				
Procedure status				
Elective	412 (89.2%)	438 (92.2%)	850 (90.7%)	0.11
Urgent	50 (10.8%)	37 (7.8%)	87 (9.3%)	
Type of valve				
Balloon-expandable	253 (54.8%)	249 (52.4%)	502 (53.6%)	0.02
Self-expandable	193 (41.8%)	221 (46.5%)	414 (44.2%)	
Valve-in-valve	13 (2.8%)	30 (6.4%)	43 (4.62%)	0.01
Vascular access site				
Femoral artery	325 (70.3%)	363 (76.4%)	688 (73.4%)	0.05
Other	129 (27.9%)	109 (22.9%)	238 (25.4%)	
Procedural complications				
Delirium	54 (11.7%)	26 (5.5%)	80 (8.5%)	< 0.001
Permanent pacemaker	70 (15.2%)	56 (11.8%)	126 (13.4%)	0.13
Stroke	10 (2.2%)	7 (1.5%)	17 (1.8%)	0.43
Bleeding/vascular complication/transfusion	176 (38.1%)	122 (25.7%)	298 (31.8%)	< 0.001
Echocardiographic findings post-TAVR				
Mitral regurgitation				
Nil/trace	133 (28.8%)	178 (37.5%)	311 (33.2%)	0.04
Mild	207 (44.8%)	195 (41.1%)	402 (42.9%)	
Moderate/Severe	104 (22.5%)	87 (18.3%)	191 (20.4%)	
Aortic regurgitation				
Nil/trace	230 (49.8%)	277 (58.3%)	507 (54.1%)	0.06
Mild	164 (35.5%)	139 (29.3%)	303 (32.3%)	
Moderate/Severe	53 (11.5%)	49 (10.3%)	102 (10.9%)	

### Mortality and causes of readmission

**Fig. 2:**
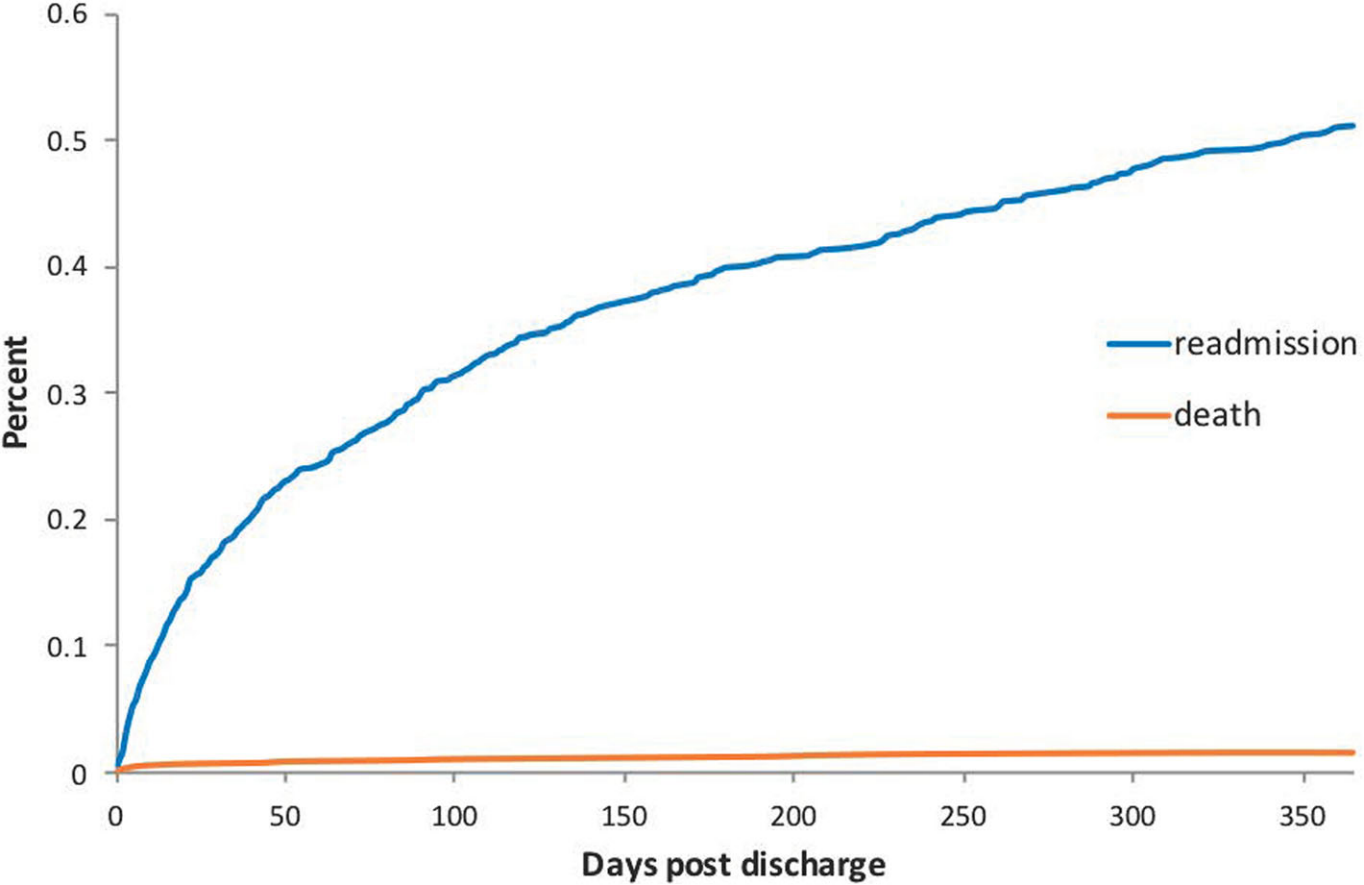
Cumulative incidence curves for readmission and death without readmission. Readmission is shown in blue and death is shown in orange

**Fig. 3:**
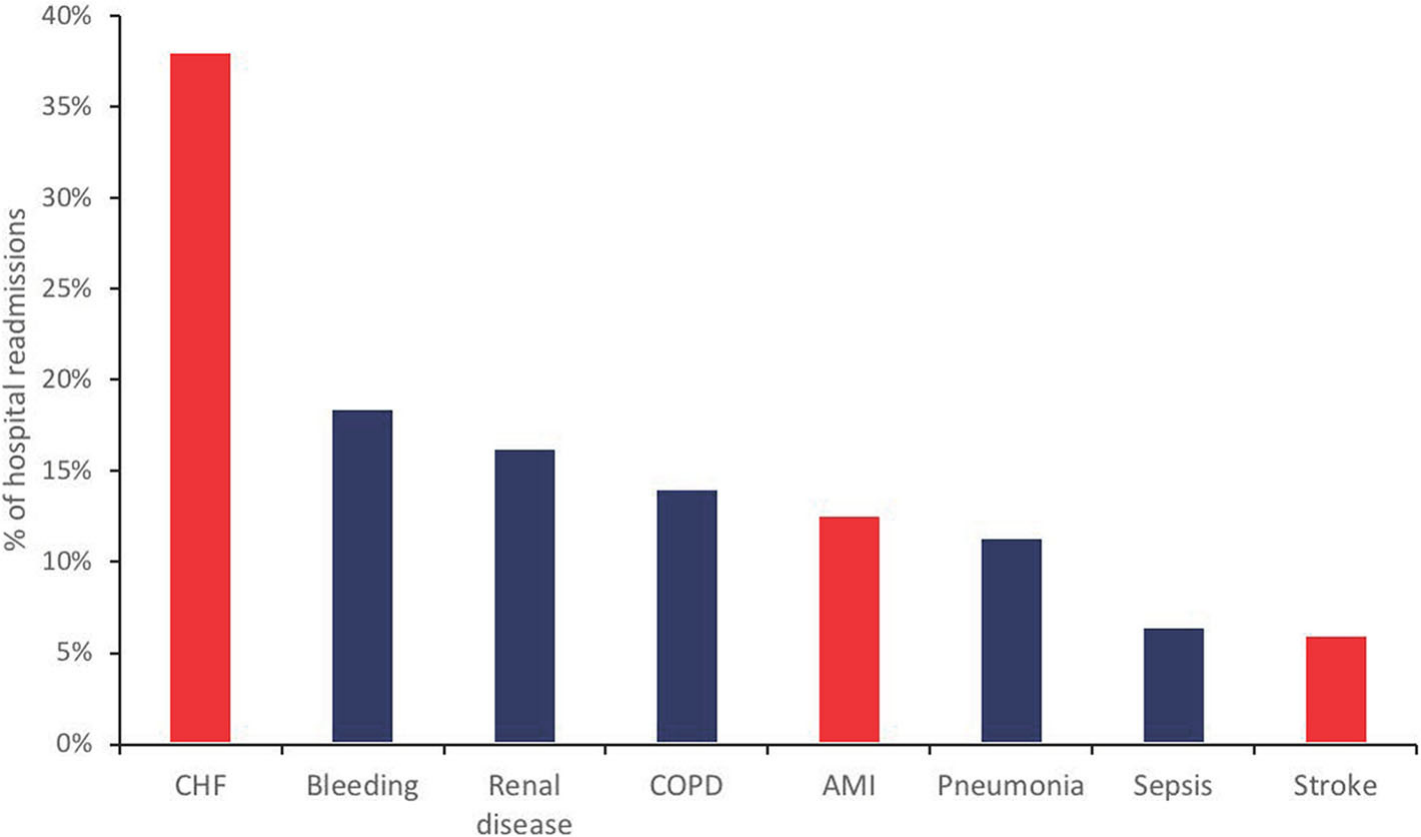
Diagnoses of patients readmitted within 1-year of TAVR. Readmission diagnoses based on most-responsible diagnosis in the year following the index TAVR hospitalization (red bars represent cardiac diagnoses and blue bars represent non-cardiac diagnoses)

At 1 year, 126 patients (13.4%) had died since discharge from the index hospitalization (Fig. 2). The majority of patients who died in the year after TAVR did so during a readmission event. The rate of 30-day readmission was 16.8% (157 patients) and the 1-year readmission rate was 49.3% (462 patients). The causes of readmission are shown in Fig. 3. The top readmission diagnoses at 1 year were heart failure and bleeding.

### Transitional care factors

None of the transitional care factors were associated with a reduction in readmission. In-patient rehabilitation (HR 1.34, 95% CI 1.11–1.62; *p* = 0.002) and cardiologist follow-up (HR 1.41, 95% CI 1.14–1.75; p = 0.002) were both associated with higher readmission rates. Home care services (HR 1.18, 95% CI 0.96–1.44; *p* = 0.12) and follow-up with a family physician (HR 1.04, 95% CI 0.85–1.28; *p* = 0.71) were not associated with the rate of hospital readmission.

### Predictors of readmission

The results of the multivariable model are shown in Table 3. The only pre-existing cardiac condition associated with readmission was atrial fibrillation with a HR of 1.34 (95% CI 1.08–1.66; *p* = 0.01), while previous myocardial infarction, heart failure and prior PCI were not. Most medical comorbidities were not significantly associated with the rate of readmission – only peripheral vascular disease (HR 1.18, 95% CI 1.02–1.37; *p* = 0.02), peptic ulcer disease (HR 1.52, 95% CI 1.09–2.12, *p* = 0.01) and dialysis (HR 1.67, 95% CI 1.06–2.65; *p* = 0.03) were associated with readmission.

**Table 3:** Predictors of hospital readmission within 1 year of TAVR discharge

	HR	95% CI	*p* value
Demographics			
Age	1.01	(1.0–1.02)	0.20
Female sex	0.72	(0.53–0.98)	0.03
Dependent living	1.03	(0.85–1.24)	0.77
Clinical Characteristics			
NYHA class			
I/II	reference		
III	1.00	(0.82–1.21)	0.96
IV	1.43	(1.07–1.91)	0.02
Frailty	1.07	(0.76–1.50)	0.69
Cardiac comorbidities and interventions			
Atrial fibrillation	1.34	(1.08–1.66)	0.01
Prior heart failure	1.08	(0.81–1.43)	0.60
Prior myocardial infarction	0.98	(0.78–1.22)	0.84
PCI	1.18	(0.92–1.52)	0.20
CABG	0.90	(0.70–1.15)	0.41
Medical comorbidities			
Diabetes	1.05	(0.86–1.29)	0.65
Cerebrovascular disease	0.82	(0.56–1.18)	0.28
Peripheral vascular disease	1.18	(1.02–1.37)	0.02
Dementia	1.22	(0.69–2.15)	0.50
Dialysis	1.67	(1.06–2.65)	0.03
Lung disease	1.23	(0.91–1.66)	0.18
Liver disease	2.00	(0.63–6.37)	0.24
Peptic ulcer disease	1.52	(1.09–2.12)	0.01
History of bleeding	1.10	(0.81–1.51)	0.54
Laboratory markers			
eGFR	1.00	(0.99–1.0)	0.36
Hemoglobin	0.99	(0.99–1.0)	< 0.001
Echocardiographic findings			
Left ventricular dysfunction	0.73	(0.54–0.98)	0.04
Procedural characteristics			
Urgent procedure	0.98	(0.58–1.65)	0.94
Valve in valve	0.65	(0.48–0.87)	0.004
Self-expandable prosthesis	0.98	(0.84–1.14)	0.76
Non-femoral vascular access site	1.40	(1.08–1.81)	0.01
TAVR year	0.97	(0.92–1.03)	0.31
Procedural complications			
Stroke	1.09	(0.82–1.45)	0.56
Permanent pacemaker	1.21	(0.83–1.77)	0.31
Delirium	1.24	(0.83–1.85)	0.29
Bleeding, vascular complication or transfusion	1.33	(1.18–1.50)	< 0.001
Echocardiographic findings post-TAVR			
Mitral regurgitation			
Nil/trace	reference		
Mild	1.23	(1.10–1.38)	< 0.001
Moderate/Severe	1.26	(1.03–1.54)	0.03
Aortic regurgitation			
Nil/trace	reference		
Mild	1.21	(0.97–1.52)	0.10
Moderate/Severe	1.13	(0.75–1.70)	0.57
Previous health resource utilization			
Hospitalization 30 days before TAVR	1.05	(0.92–1.20)	0.48
Transitional care factors			
Rehabilitation	1.34	(1.11–1.62)	0.002
Home care	1.18	(0.96–1.44)	0.12
Family physician follow-up	1.04	(0.85–1.28)	0.71
Cardiologist follow-up	1.41	(1.14–1.75)	0.002

Abbreviations: *NYHA* New York Heart Association, *PCI* percutaneous coronary intervention, *CABG* coronary-aorto bypass grafting

Procedural characteristics and complications had inconsistent effects on readmission. Valve-in-valve procedures were strongly associated with a lower rate of readmission (HR 0.65, 95% CI 0.48–0.87, *p* = 0.004), while non-femoral vascular access was a significant predictor of higher readmission with a HR of 1.40 (95% CI 1.08–1.81; p = 0.01). The year of the TAVR procedure did not influence readmission.

### Sensitivity analyses

After exclusion of patients who died or were readmitted in the first 30 days, there were 774 patients available for the landmark analysis (Appendix 1). The influence of transitional care factors on readmission remained the same for in-patient rehabilitation, home care, and family physician follow-up. However, the influence of cardiologist visits changed significantly, as there was no longer an increased hazard for readmission (HR 1.13, 95% CI 0.89–1.42) associated with follow-up in the 30 days following discharge from the index hospitalization.

There were 677 patients who underwent TAVR after 2010. The overall results of this model are consistent with the primary model, suggesting that change in procedural care over time did not significantly influence hospital readmission (Appendix 2).

## Discussion

We demonstrated that nearly half of patients who underwent TAVR were readmitted to hospital within 1 year, while the most common causes of readmission were heart failure and bleeding. Moreover, transitional care factors including home care, rehabilitation and physician follow-up were not associated with a reduced rate of readmission and some were associated with potentially higher hazard of readmission. While these factors cannot be endorsed for targeted improvement efforts based on our data, other modifiable targets that may potentially reduce readmission include use of bleeding avoidance strategies and optimal heart failure care.

Physician follow-up has been associated with a reduced risk of readmission after hospitalization in a variety of settings, including heart failure [4–7]. We were not able to replicate these results in the TAVR population despite high rates of pre-existing heart failure and readmission. There are several potential reasons for these divergent findings. First, our findings could be influenced by ‘bias by indication’. Patients who receive earlier follow-up, and in general, more attention from healthcare providers, are likely to have more comorbid conditions, more complications, poor social supports and poor functional abilities. Although we designed a robust statistical model with the inclusion of wide-ranging patient and procedural characteristics, it is possible that this finding is a reflection of residual confounders. Secondly, our study design utilized an analytical approach centered around the use of time-varying covariates for transitional care factors. While this allowed us to avoid misclassification bias, it may have led to a ‘bias’ towards early and unplanned visits (as opposed to routine follow-up) which may have led to readmission. The fact that the landmark analysis did not show an association between follow-up and readmission supports these conclusions. Finally, certain conditions may be more sensitive to post-discharge care than others. Optimal heart failure management involves close follow-up for titration of medications, patient education, and monitoring weights, which adds face validity to follow-up as a tool for reducing readmissions. In contrast, close follow-up in complex medical patients with multiple comorbidities has not been associated with reduced readmission, even when combined with intense out-patient support structures and educational initiatives [19, 20]. This suggests that while certain discrete conditions may be sensitive to close follow-up care, the heterogeneity of the TAVR population may have obscured any potential benefit.

The leading cause of hospital readmission after TAVR was heart failure and optimization of heart failure management may be an important component of quality improvement strategies. Multi-disciplinary heart function clinics which assist in transition of care have been deemed an essential element of heart failure care [21]. Identification of patients who are at highest risk, followed by targeted implementation of such a model, may lead to improved outcomes. Some of our study findings may support this assertion, in that we found left ventricular dysfunction to be associated with a lower risk of readmission. We hypothesize that this may be related to more careful attention to heart failure management in these patients. Future research should seek to identify which patients are at highest risk for heart failure readmission and whether these factors are modifiable, ideally through a randomized trial.

Bleeding has emerged as a major cause of readmission after TAVR [2]. It has also been shown to increase mortality well beyond the peri-procedural period and is most often gastrointestinal in origin [22]. We found several independent predictors of readmission that are all associated with bleeding, including: atrial fibrillation, peptic ulcer disease, baseline hemoglobin and bleeding complications during the index hospitalization. These findings suggest that assessment of bleeding risk and application of bleeding avoidance strategies have the potential to alter readmission rates. Several strategies could be explored in this regard including formal bleeding risk assessment (such as HAS-BLED), prescription of proton-pump inhibitors and routine optimization and work-up of pre-procedural anemia [23, 24]. Finally, these findings suggest that a risk-benefit approach should be used when prescribing anti-platelet and anti-coagulant medication to patients after TAVR, acknowledging that there is currently limited evidence to guide practice.

Several limitations of our work warrant acknowledgement. Frist, transitional care factors are complex, and their influence on hospital readmission can be difficult to study in an observational study. To mitigate this, we used a sophisticated analytical model to account for confounding factors. Since randomized control trials are unlikely to be performed in this area, our results should act to enhance our understanding of TAVR readmission. Second, our cohort included patients who underwent TAVR between 2007 and 2013, which may be criticized for being too far removed from contemporary practice. To address this concern, we performed a sensitivity analysis that included patients from 2011 to 2013 and demonstrated results consistent with our primary model. We also included procedure year as a predictor in our models and found it to be non-significant. Furthermore, since current readmission rates are in keeping with those observed in our study, we anticipate that the overall etiologic insights we have been able to generate are still applicable to current practice [3, 25].

## Conclusion

Hospital readmission after TAVR has emerged as a major challenge, further exacerbated by growing demand. Heart failure and bleeding are important causes of morbidity in this population, suggesting that targeted interventions at these conditions might alter readmission rates. Finally, transitional care factors were not associated with a reduced rate of readmission after TAVR and these results do not support a focus on these as readmission-reducing transitional care interventions. Nonetheless, they should also not be used to dissuade clinicians from best practices surrounding hospital discharge. Further studies are needed to assess whether formally structured or targeted discharge interventions, are effective at altering readmission patterns in this population.

## Appendix 1

**Table 4:** Landmark Analysis: Predictors of readmission from 31-365 days after discharge

	HR	95% CI	*p* value
Demographics			
Age	1.00	(0.99 - 1.01)	0.67
Female sex	0.92	(0.61 - 1.40)	0.70
Dependent living	0.93	(0.72 - 1.20)	0.56
Clinical Characteristics			
NYHA class			
I/II	reference		
III	1.15	(0.83 - 1.61)	0.40
IV	1.68	(1.06 - 2.66)	0.03
Frailty	0.87	(0.66 - 1.13)	0.30
Cardiac comorbidities and interventions			
Atrial fibrillation	1.30	(1.02 - 1.65)	0.03
Prior heart failure	1.02	(0.83 - 1.26)	0.84
Prior myocardial infarction	0.97	(0.68 - 1.39)	0.87
PCI	1.02	(0.73 - 1.43)	0.92
CABG	0.96	(0.76 - 1.22)	0.73
Medical comorbidities			
Diabetes	0.96	(0.63 - 1.48)	0.86
Cerebrovascular disease	0.70	(0.50 - 0.98)	0.04
Peripheral vascular disease	1.27	(1.02 - 1.57)	0.03
Dementia	1.03	(0.60 - 1.78)	0.90
Dialysis	2.01	(0.90 - 4.48)	0.09
Lung disease	1.33	(1.08 - 1.65)	0.01
Liver disease	1.52	(0.43 - 5.36)	0.51
Peptic ulcer disease	1.86	(1.29 - 2.67)	<0.001
History of bleeding	1.08	(0.79 - 1.47)	0.63
Laboratory markers			
eGFR	0.99	(0.99 - 1.00)	0.14
Hemoglobin	0.99	(0.99 - 1.00)	<0.001
Echocardiographic findings			
Left ventricular dysfunction	0.76	(0.57 - 1.02)	0.07
Procedural characteristics			
Urgent procedure	1.10	(0.66 - 1.81)	0.72
Valve in valve	0.49	(0.29 - 0.83)	0.01
Self-expandable prosthesis	1.15	(0.89 - 1.48)	0.29
Non-femoral vascular access site	1.17	(0.78 - 1.75)	0.44
TAVR year	0.99	(0.90 - 1.10)	0.90
Procedural complications			
Stroke	1.78	(1.22 - 2.60)	0.003
Permanent pacemaker	0.97	(0.53 - 1.76)	0.91
Delirium	1.40	(0.95 - 2.06)	0.09
Bleeding, vascular complication or transfusion	1.29	(1.22 - 1.37)	<0.001
Echocardiographic findings post-TAVR			
Mitral regurgitation			
Nil/trace	reference		
Mild	1.06	(0.95 - 1.20)	0.30
Moderate/Severe	1.27	(0.97 - 1.66)	0.08
Aortic regurgitation			
Nil/trace	reference		
Mild	1.25	(0.95 - 1.64)	0.11
Moderate/Severe	1.29	(0.77 - 2.15)	0.34
Previous health resource utilization			
Hospitalization 30 days before TAVR	1.21	(0.91 - 1.61)	0.20
Transitional care factors in first 30 days			
Rehabilitation	1.55	(1.37 - 1.75)	<0.001
Home care	0.94	(0.78 - 1.13)	0.51
Family physician follow-up	0.73	(0.51 - 1.05)	0.09
Cardiologist follow-up	1.13	(0.89 - 1.42)	0.32

Abbreviations: *NYHA* New York Heart Association, *PCI* percutaneous coronary intervention, *CABG* coronary-aorto bypass grafting

## Appendix 2

**Table 5:** Contemporary Cohort (after 2010): Predictors of hospital readmission within 365 days of discharge

	HR	95% CI	*p* value
Demographics			
Age	1.00	(1.00 - 1.01)	0.49
Female sex	0.80	(0.51 - 1.24)	0.32
Dependent living	0.90	(0.64 - 1.26)	0.53
Clinical Characteristics			
NYHA class			
I/II	reference		
III	1.03	(0.78 - 1.34)	0.85
IV	1.36	(0.99 - 1.87)	0.06
Frailty	0.92	(0.64 - 1.33)	0.67
Cardiac comorbidities and interventions			
Atrial fibrillation	1.29	(1.02 - 1.63)	0.04
Prior heart failure	1.12	(0.74 - 1.69)	0.59
Prior myocardial infarction	1.03	(0.73 - 1.44)	0.88
PCI	1.17	(0.84 - 1.64)	0.35
CABG	0.80	(0.57 - 1.12)	0.19
Medical comorbidities			
Diabetes	0.97	(0.78 - 1.19)	0.74
Cerebrovascular disease	0.75	(0.48 - 1.17)	0.21
Peripheral vascular disease	1.38	(1.12 - 1.70)	0.003
Dementia	1.32	(0.56 - 3.08)	0.53
Dialysis	1.75	(1.18 - 2.59)	0.01
Lung disease	1.15	(0.66 - 1.98)	0.62
Liver disease	1.58	(0.22 - 11.2)	0.65
Peptic ulcer disease	1.52	(1.01 - 2.27)	0.04
History of bleeding	1.09	(0.72 - 1.66)	0.69
Laboratory markers			
eGFR	1.00	(0.99 - 1.00)	0.19
Hemoglobin	0.99	(0.99 - 1.00)	<0.001
Echocardiographic findings			
Left ventricular dysfunction	0.78	(0.59 - 1.04)	0.09
Procedural characteristics			
Urgent procedure	1.08	(0.64 - 1.84)	0.77
Valve in valve	0.76	(0.59 - 0.99)	0.04
Self-expandable prosthesis	0.94	(0.82 - 1.07)	0.36
Non-femoral vascular access site	1.38	(1.05 - 1.81)	0.02
TAVR year	0.92	(0.82 - 1.04)	0.18
Procedural complications			
Stroke	1.27	(0.80 - 2.02)	0.32
Permanent pacemaker	1.43	(0.92 - 2.22)	0.11
Delirium	1.26	(0.87 - 1.85)	0.23
Bleeding, vascular complication or transfusion	1.38	(1.13 - 1.68)	0.001
Echocardiographic findings post-TAVR			
Mitral regurgitation			
Nil/trace	reference		
Mild	1.23	(0.86 - 1.76)	0.26
Moderate/Severe	1.29	(0.89 - 1.86)	0.17
Aortic regurgitation			
Nil/trace	reference		
Mild	1.24	(1.05 - 1.47)	0.01
Moderate/Severe	1.35	(0.95 - 1.93)	0.10
Previous health resource utilization			
Hospitalization 30 days before TAVR	1.09	(0.88 - 1.35)	0.44
Transitional care factors			
Rehabilitation	1.34	(1.12 - 1.61)	0.002
Home care	1.12	(0.85 - 1.48)	0.42
Family physician follow-up	0.93	(0.69 - 1.25)	0.63
Cardiologist follow-up	1.29	(1.05 - 1.58)	0.02

Abbreviations: *NYHA* New York Heart Association, *PCI* percutaneous coronary intervention, *CABG* coronary-aorto bypass grafting

## Abbreviations

AS: Aortic stenosis
NYHA: New York Heart Association
PCI: Percutaneous coronary intervention
TAVR: Trans-catheter aortic valve replacement
